# Serum Soluble Interleukin-2 Receptor Does Not Differentiate Complex Regional Pain Syndrome from Other Pain Conditions in a Tertiary Referral Setting

**DOI:** 10.1155/2020/6259064

**Published:** 2020-09-28

**Authors:** K. D. Bharwani, M. Dirckx, D. L. Stronks, W. A. Dik, F. J. P. M. Huygen

**Affiliations:** ^1^Center for Pain Medicine, Department of Anesthesiology, Erasmus MC University Medical Center Rotterdam, Netherlands; ^2^Laboratory Medical Immunology, Department of Immunology, Erasmus MC University Medical Center Rotterdam, Netherlands; ^3^Department of Internal Medicine, Division of Clinical Immunology, Erasmus MC University Medical Center Rotterdam, Netherlands

## Abstract

Previously, we showed that serum soluble interleukin-2 receptor (sIL-2R) levels, a marker for T-cell activation, were higher in complex regional pain syndrome (CRPS) patients than in healthy controls, suggesting pathogenic T-cell activation in CRPS. Additionally, sIL-2R levels discriminated well between CRPS and healthy controls with a high sensitivity (90%) and specificity (89.5%), suggesting a possible role for sIL-2R in the diagnosis of CRPS. In order to further validate this marker in the diagnostic workup of CRPS, we conducted this prospective cohort study in which we determined sIL-2R levels in patients that were referred to our tertiary referral center with a suspicion of CRPS in a limb, and subsequently compared sIL-2R levels between the patients that were diagnosed with CRPS (CRPS group) and those who were not (no CRPS group). A group of anonymous blood bank donors were used as a healthy control group. Furthermore, we explored the relationship between sIL-2R and CRPS disease severity using the CRPS severity score. Median sIL-2R levels of both the CRPS group (2809.0 pg/ml; Q3-Q1: 3913.0-1589.0) and no CRPS group (3654.0 pg/ml; Q3-Q1: 4429.0-2095.5) were significantly higher than that of the control group (1515.0 pg/ml; Q3-Q1: 1880.0-1150.0): CRPS vs. controls, *p* < .001; no CRPS vs. controls, *p* < 0.001. Serum sIL-2R levels did not differ significantly between the CRPS and no CRPS group. A statistically significant negative correlation was observed between sIL-2R levels and the CRPS severity score (*r*_s_ = −0.468, *p* = 0.024). Our results confirm our previous findings of higher sIL-2R levels in CRPS patients than in healthy controls. We further showed that serum sIL-2R cannot differentiate between CRPS and other pain conditions of a limb in a tertiary referral setting. Interestingly, a negative correlation was found between sIL-2R and CRPS disease severity; this finding warrants further research into the relationship between sIL-2R and CRPS disease severity.

## 1. Introduction

Complex regional pain syndrome (CRPS) is characterized by continuous pain which is accompanied by various sensory, motor, vasomotor, sudomotor, and trophic disturbances [1]. The onset of CRPS is preceded by damage to the tissues of a limb, for example, due to fracture or surgery [2]. If CRPS is left untreated, it can have incapacitating consequences not only on the function of the affected limb, but also on the social life of patients [3]. However, appropriate treatment is often initiated too late due to a delay in diagnosis [4].

This diagnostic delay is mostly due to two reasons. First, the diagnosis of CRPS is still based on a set of relatively subjective criteria: the New International Association for the Study of Pain clinical diagnostic criteria for CRPS [1]. Thus, the (early) diagnosis of CRPS cannot yet be established by objective diagnostic testing. Second, the pathophysiology of CRPS is complex and still incompletely understood; this lack of understanding creates skepticism among physicians on whether this disease exists [5, 6] and further leads to a general lack of awareness on the symptoms and signs of this disease.

Although the pathophysiology of CRPS is still incompletely understood, it has been established that it comprises of multiple disease mechanisms [7]. Inflammation is recognized as one of the pathophysiological mechanisms contributing to CRPS. This inflammation may, in part, be related to dysregulation of the immune system associated with altered T-cell activity [8–10]. Our group previously assessed T-cell activity in CRPS patients by measuring serum levels of the soluble interleukin-2 receptor (sIL-2R): a marker for T-cell activation [8, 11, 12]. We found significantly higher serum sIL-2R levels in the CRPS group than in healthy controls, supporting the notion of pathological T-cell activation in CRPS [8]. Moreover, serum sIL-2R level discriminated well between CRPS patients and healthy controls, with a high sensitivity (90%) and specificity (89.5%) [8].

This last finding is especially noteworthy as it indicates that serum sIL-2R may represent a biomarker to facilitate the diagnosis of CRPS. Elevated serum sIL-2R levels are, however, not disease specific as this is found in many different disease entities, including immune and rheumatic diseases, as well as malignancies [13]. Yet, the potential diagnostic value of serum sIL-2R was recently demonstrated in a retrospective cohort study in patients suspected of sarcoidosis [14]. On the basis of an established cut-off value, the sensitivity and specificity of serum sIL-2R for the detection of sarcoidosis were 88% and 85%, by far superior to angiotensin-converting enzyme (ACE; the classical biomarker for sarcoidosis with a sensitivity of 62% and specificity of 88%) [14]. Therefore, we consider it of interest to further explore the potential application of serum sIL-2R measurement in establishing the diagnosis of CRPS. At this moment, biomarkers validated for use in the diagnosis of CRPS are not available. However, identification of potential diagnostic biomarkers could greatly aid in preventing a delayed diagnosis and starting appropriate and timely therapy in CRPS.

Previously, we determined serum sIL-2R levels only in CRPS patients and healthy controls and consequently, we could not draw conclusions on the role of serum sIL-2R in the diagnostic workup of CRPS [8]. Therefore, in this current study, we examined whether serum sIL-2R can be used to differentiate CRPS from other pain conditions of a limb in patients referred to a tertiary referral center due to a suspicion of CRPS.

## 2. Materials and Methods

### 2.1. Ethical Approval

This study was conducted according to the principles of the Declaration of Helsinki and in accordance with the Medical Research Involving Human Subjects Act (WMO). The study was approved by the Medical Ethics Committee of Erasmus MC University Medical Center Rotterdam (MEC-2017-495). The trial was registered in the Netherlands Trial Registry (NTR7465).

### 2.2. Study Design, Recruitment, and Study Population

This prospective cohort study was conducted at the Center for Pain Medicine (CPM) at Erasmus MC University Medical Center which is a teaching hospital located in Rotterdam, the Netherlands. The CPM is a tertiary referral center with CRPS being one of the fields of expertise. Patients are referred to our center by general physicians or other specialists such as orthopedic surgeons.

All patients referred to our center with a suspicion of CRPS in one limb were invited to participate in this study. Two weeks before their first outpatient clinic appointment, patients were approached by a study physician with both verbal and written information on the study. The patients could decide on the day of their appointment whether they wanted to participate in the study. Patients were informed that the results of this study would not influence the diagnosis or treatment of their disease. After obtaining informed consent, the inclusion and exclusion criteria described in Table 1 were applied. Patients were included consecutively until the required sample size was reached. The inclusion period started in March 2018 and ended in August 2019.

Serum sIL-2R levels available from 101 anonymous healthy blood bank donors served as a reference for serum sIL-2R levels in the healthy population. Thus, the study population consisted of 3 groups: patients finally diagnosed with CRPS (CRPS group), patients finally diagnosed with a condition other than CRPS (no CRPS group), and healthy controls.

### 2.3. Study Measurements and Data Collection

The following data were collected during the outpatient clinic appointment: age; duration of disease (i.e., duration of symptoms and signs); precipitating injury (i.e., initiating factor of symptoms and signs); affected limb; medication; intensity of pain at the moment of the visit and in the past 24 hours using an 11-point numeric rating scale (NRS); and symptoms and signs recorded using the CRPS severity score-Database Form developed by Harden et al. along with the resulting CRPS severity score (CSS) [15] (Table 2). Permission was received from N. Harden for use of the CRPS severity score-Database Form [15]. The study physicians followed the instructions of the CRPS severity score-Database Form to register symptoms and signs during physical examination. At the end of the appointment, one 5-milliliter tube of venous blood was drawn for sIL-2R analysis.

### 2.4. Diagnosis of CRPS Group and No CRPS Group

CRPS was diagnosed using the widely accepted New International Association for the Study of Pain clinical diagnostic criteria for CRPS [1]. All other diagnoses were established using appropriate and up-to-date guidelines, and when needed, patients were referred to the appropriate specialty. The diagnoses of patients in the no CRPS group were divided into the following categories: neuropathic pain syndromes, myofascial pain syndromes, vascular diseases, inflammatory conditions, and psychiatric problems/disorders. These categories were derived from the differential diagnosis of CRPS as described in the article by van Eijs et al. [16].

### 2.5. sIL-2R Analysis

Venous blood samples were centrifuged at 3000 rpm after collection, and serum was subsequently isolated. Soluble IL-2R levels were measured using an enzyme-linked immunosorbent assay (Human sCD25/sIL-2R ELISA kit, Besancon, Cedex, France) according to the manufacturer's instructions at the diagnostic Laboratory Medical Immunology facility of Erasmus MC University Medical Center Rotterdam. The measurements were conducted under strict quality procedures (ISO15189).

### 2.6. Sample Size Calculation

Based on the results of our previous study [8], we chose a statistically detectable and clinically relevant effect size (*d*) of 1.0 on serum sIL-2R level using an independent *t*-test. The power of the study (1-*β*) was set at 0.8, the allocation ratio at 0.25, and the two-sided level of significance (*α*) at 0.05. The required sample size computed by this method was 52.

### 2.7. Statistical Analysis

Descriptive statistics were used to calculate the frequencies of categorical variables and to calculate measures of central tendency and variability of continuous variables. The Shapiro-Wilk test was used to analyze whether continuous variables were normally distributed. Variables with a skewed distribution are reported in medians and interquartile ranges (Q3-Q1), otherwise means and standard deviations are used. The primary outcome parameter was the serum sIL-2R level in the CRPS group, no CRPS group, and healthy control group.

Depending on the shape of distribution, continuous variables were compared between two groups using either a two-sided independent *t*-test or a two-sided Mann-Whitney *U* test. Comparison of continuous variables between more than two groups was conducted using either an ANOVA or a Kruskal-Wallis test, dependent on the shape of the distribution of the variable. Categorical variables were compared using the Fisher's exact test.

A possible association in the CRPS group between sIL-2R levels and age, sIL-2R levels and duration of disease, and sIL-2R levels and the CRPS severity score was explored using either a Pearson's correlation or a Spearman's rank correlation, dependent on the shape of the distribution of these variables. A possible association in the CRPS group between sIL-2R levels and gender was explored using a point-biserial correlation.

Where possible, data are presented in tables and graphs, such as box-and-whisper plots and scatterplots. For box-and-whisper plots that are created in SPSS, the box represents the interquartile range and the whiskers extend to the highest and lowest value in the data range which are no greater than 1.5 times the interquartile range. Circles in the box-and-whisker plots indicate outliers that are between 1.5 and 3 times the interquartile range. Analyses were performed using IBM SPSS Statistics 21. The alpha level for statistical significance was set at 0.05.

## 3. Results

**Figure 1:**
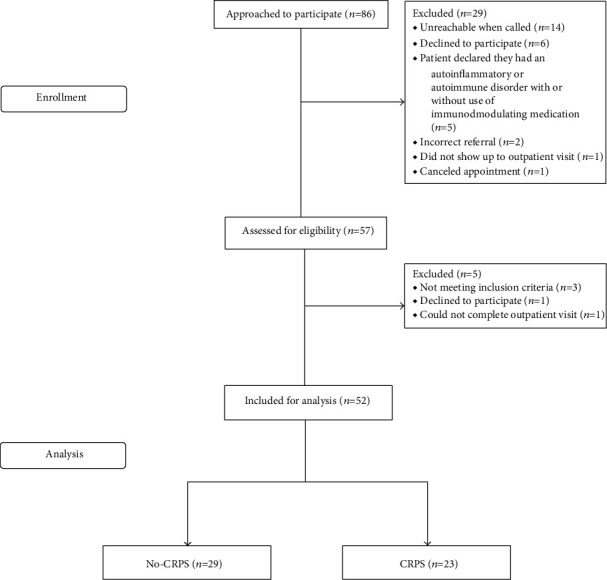
Flow diagram depicting the recruitment and inclusion of the study population.

Figure 1 depicts the recruitment and inclusion of our study population. A total of 86 patients were approached to participate in this study. Twenty-nine patients did not participate in the study: one patient canceled the outpatient clinic appointment; one patient did not show up at the appointment; two patients had an incorrect referral; five patients declared, of their own accord, during the phone call that they have an autoimmune or autoinflammatory disorder with or without use of immunomodulating medication; six patients were unwilling to participate in research; fourteen patients were unreachable when called. Fifty-seven patients signed the informed consent form. Five patients were excluded after signing the form: two patients were excluded due to use of prednisolone; one patient did not have time to complete the outpatient visit; one patient backed out without further explanation; one patient was excluded because of a history of active psoriasis. This resulted in the required sample size of 52 patients for analysis.

Of the 52 patients, 23 patients (44%) were diagnosed with CRPS and 29 patients (56%) were diagnosed with other conditions (no CRPS group). Of the no CRPS group, 7 patients (24.1%) were diagnosed with neuropathic pain syndromes, 17 (58.6%) with myofascial pain syndromes, 2 patients (6.9%) with inflammatory conditions, and 3 patients (10.3%) had an unclear or unknown diagnosis. No diagnoses were made that could be categorized as vascular diseases or psychiatric problems/disorders. Full details of the no CRPS group, including diagnoses that were made per category, can be found in Table 3.

Patient characteristics such as age, gender, affected limb, precipitating injury, and duration of disease were comparable between both the CRPS group and the no CRPS group (Table 4). Use of medication was also comparable between both groups (Table 5). Median pain scores at the time of visit and 24 hours before the visit were also comparable between both groups (Table 6).

Table 6 shows the proportion of symptoms and signs in each group recorded according to the CRPS severity score-Database Form [15]. The prevalence of the following symptoms (i.e., subjective symptoms reported by patients) was significantly higher in the CRPS group than in the no CRPS group: continuing pain, color asymmetry, and decreased active range of motion of the affected limb. The prevalence of the following signs (i.e., objective signs observed by the physician) was significantly higher in the CRPS group than in the no CRPS group: hyperalgesia to pinprick; allodynia and its corresponding subcategories; temperature asymmetry, with all affected CRPS patients having a cooler affected limb; color asymmetry and its corresponding subcategory “red”; sweating asymmetry, with all affected CRPS patients experiencing increased sweating on the affected side; and asymmetric edema. The mean CRPS severity score was significantly higher in the CRPS group than in the no CRPS group (CRPS 11.4 (sd = 2.2) versus no CRPS 8.1 (sd = 1.9), *p* < 0.001).

**Figure 2:**
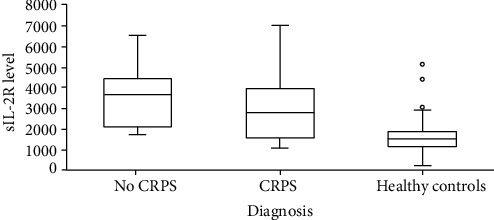
Boxplot of the median sIL-2R levels in the no CRPS group (3654.0 pg/ml; Q3-Q1: 4429.0-2095.5), the CRPS group (2809.0 pg/ml; Q3-Q1: 3913.0-1589.0), and the control group (1515.0 pg/ml; Q3-Q1: 1880.0-1150.0): CRPS vs. controls, *p* < .001 and no CRPS vs. controls, *p* < 0.001.

The median sIL-2R levels of both the CRPS group (2809.0 pg/ml; Q3-Q1: 3913.0-1589.0) and no CRPS group (3654.0 pg/ml; Q3-Q1: 4429.0-2095.5) were significantly higher than the median sIL-2R level of the control group (1515.0 pg/ml; Q3-Q1: 1880.0-1150.0): CRPS group vs. control group, *p* < .001 and no CRPS group vs. control group, *p* < 0.001. Serum sIL-2R levels did not differ significantly between the CRPS group and no CRPS group (Figure 2 and Table 3).

**Figure 3:**
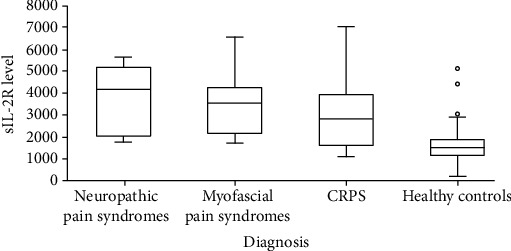
Boxplot of median sIL-2R levels in the neuropathic pain syndrome group (4170.0 pg/ml; Q3-Q1: 5203.0-2050.0), the myofascial pain syndrome group (3529.0 pg/ml; Q3-Q1: 4253.5-2150.5), the CRPS group (2809.0 pg/ml; Q3-Q1: 3913.0-1589.0), and the group of healthy controls (1515.0 pg/ml; Q3-Q1: 1880.0-1150.0): neuropathic pain syndromes versus controls, *p* < 0.001 and myofascial pain syndromes versus controls, *p* < 0.001.

Of the no CRPS group, both the neuropathic pain syndrome group (4170.0 pg/ml; Q3-Q1: 5203.0-2050.0) and myofascial pain syndrome group (3529.0 pg/ml; Q3-Q1: 4253.5-2150.5) had median sIL-2R levels that were significantly higher than the median sIL-2R level of healthy controls (1515.0 pg/ml; Q3-Q1: 1880.0-1150.0): neuropathic pain syndrome group versus control group, *p* < 0.001 and myofascial pain syndrome group versus control group, *p* < 0.001. There was no significant difference in the distribution of sIL-2R levels between the neuropathic pain syndrome group, myofascial pain syndrome group, and the CRPS group (Figure 3 and Table 3).

**Figure 4:**
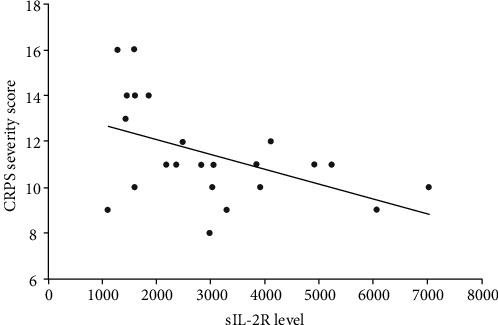
Scatter plot showing the correlation between serum sIL-2R level and CRPS severity score in CRPS patients: *r*_s_ = −0.468, *p* = 0.024.

Within the CRPS group, a statistically significant negative correlation existed between serum sIL-2R levels and the CRPS severity score (*r*_s_ = −0.468, *p* = 0.024, Figure 4). No association was found between serum sIL-2R level and age, gender, and disease duration in the CRPS group.

## 4. Discussion

So far, objective diagnostic tests to diagnose CRPS are not available. This lack of objective tests hampers early diagnosis and timely initiation of appropriate therapies [17]. Based on the findings from our previous study in which sIL-2R levels were found to be significantly higher in CRPS patients than in healthy controls [8], we conducted this current study in which we investigated whether serum sIL-2R could be used to help establish the diagnosis CRPS in patients who were referred to a tertiary referral center with pain in a limb that was suspected to be caused by CRPS. To our knowledge, this is the first study assessing the differentiating capacity of serum sIL-2R in CRPS. Our results indicate that serum sIL-2R is not useful for differentiating CRPS from other pain conditions of a limb in patients referred with a suspicion of CRPS to a tertiary referral center.

One of the main explanations why serum sIL-2R may not be useful in differentiating CRPS from other pain conditions of a limb may be that altered T-cell activity occurs in various diseases that are part of the initial differential diagnosis of CRPS. For example, there are diseases in the differential diagnosis of CRPS that have been proven to involve T-cell activation and have been shown to have elevated sIL-2R levels, such as rheumatoid arthritis [13, 18]. Recently, carpal tunnel syndrome (CTS)—which also needs to be considered in the differential diagnosis of CRPS of the upper limb—was shown to be associated with elevated percentages of central and effector memory CD4^+^ T-cells which is suggestive of changes in memory T-cell homeostasis in CTS [19]. Therefore, we consider it likely that serum sIL-2R levels may be elevated in CTS patients as well, although data on this is lacking so far. There is also evidence that altered T-cell activity may play a role in (the development of) neuropathic pain [20, 21]. It is thus plausible that there is altered T-cell activity in the various diseases that make up the differential diagnosis of CRPS, thereby diminishing any differentiating power serum sIL-2R may have in the diagnosis of CRPS. Moreover, as stated in the introduction, elevated serum sIL-2R levels are not disease specific as elevated levels of sIL-2R can be found in many different diseases [12, 13].

In this study, we have confirmed our previous finding of elevated serum sIL-2R levels in CRPS, indicating that T-cell activation is involved in the pathogenesis of CRPS [8–10]. It was further observed that the group of neuropathic pain syndromes was also associated with elevated serum sIL-2R levels, indicating that T-cell activation is likely to be involved in these pain syndromes. In line with this, recent observations in animal models support an important role for T-cells in (the development of) neuropathic pain [20, 21]. We also found significantly higher sIL-2R levels in the myofascial pain syndrome group than in the group of healthy controls. This may be related to the various diagnoses we categorized into this group. For reasons of simplicity, we categorized diseases as myofascial pain syndromes if they were not considered neuropathic or “classically inflammatory” by nature. However, it is not unthinkable that certain diseases we classified in this group, such as osteoarthritis, could reveal increased sIL-2R levels (Table 3) [22]. Nevertheless, studies in larger cohorts should separately explore the contribution of T-cells to the various diseases categorized into the group of myofascial pain syndromes.

Interestingly, we further found a statistically significant negative correlation between sIL-2R levels and the CRPS severity score in our CRPS patients. We propose three explanations for this negative correlation in our cohort of CRPS patients. First, it is possible that serum sIL-2R level reflects T-cell-driven inflammatory disease activity (the intensity of the inflammatory process) rather than disease severity (the impact of the disease activity on the limb) in CRPS. Such would indicate that serum sIL-2R level measured in CRPS may be strongly related to the phase of disease. Patients in the acute phase of CRPS often present with the warm subtype of CRPS [2, 23]. As the disease progresses and becomes chronic, most patients undergo a change from a warm (acute) subtype to a cold (chronic) subtype [24]. It is thought that this subtype transition is caused by a change in active underlying pathophysiological mechanisms during the course of this syndrome. For example, inflammatory mechanisms seem to be most prominent in the warm (acute) CRPS subtype and seem to diminish as the disease progresses [24]. However, (tissue) damage inflicted by the early inflammatory phase may persist and even worsen because of other pathophysiological mechanisms that gain the upper hand. Considering that all CRPS patients in this study had chronic CRPS, it is possible that in this group of chronic CRPS patients, T-cell-mediated inflammatory disease activity, and thus sIL-2R level, has diminished over time while the damage caused by this activity—the disease severity—remains extensive. It would be interesting to test this hypothesis with serial measurements of sIL-2R in a prospective cohort of acute CRPS patients.

Second, this negative correlation may be explained by an immunosuppressive biological function of sIL-2R. The sIL-2R is the circulating form of the *α*-chain of the membrane-bound high-affinity trimeric interleukin-2 (IL-2) receptor. IL-2 is an important regulatory cytokine for the activation, proliferation, differentiation, and survival of different T-cell subsets [12, 25, 26]. It has been suggested that circulating sIL-2R competes for available IL-2 and may limit activation and proliferation of T-lymphocytes by sequestration of available IL-2 [12, 25, 27–31]. It has further been proposed that sIL-2R presents IL-2 to CD4^+^ T-helper cells, thereby inducing T-cell differentiation towards anti-inflammatory T-regulatory cells (Tregs) instead of proinflammatory Th1 or Th17 cells [25, 32]. Considering that the discovered negative correlation suggests a higher sIL-2R is associated with less disease severity, it can be hypothesized that sIL-2R may have an immunosuppressive, and thus protective, biological function in CRPS. This idea is partially supported by the findings in the study by Heyn et al. in which the authors found a significantly lower percentage of proinflammatory Th17 cells, a lower Th17/Treg ratio, and a significantly higher proportion of anti-inflammatory CD39+Tregs in a group of CRPS patients, suggesting an anti-inflammatory T-cell shift in CRPS [9].

Third, the negative correlation may reveal an inability of our clinical observations to objectify a possible T-cell-mediated inflammatory pathology and the related disease activity and severity in CRPS. This inability of our clinical observations to reflect an underlying pathology could explain the discrepancy between biochemical changes and clinical findings that are often found in CRPS.

Thus, although in our current study serum sIL-2R seems to lack diagnostic value when it comes to differentiating CRPS from other pain conditions of a limb with a similar presentation, it seems that this marker may have a potential role in the monitoring of disease activity and/or severity of CRPS. This warrants future research in which the relationship between serum sIL-2R levels and disease activity and severity of CRPS are explored.

We made two interesting observations in this study: first, our current study population had a relatively long disease duration; second, at the time of measurement, patients who suffered from temperature changes all had a cool limb. Our Center for Pain Medicine is a tertiary referral center, and it seems that the cases that are referred to us are usually the cases that are refractory to therapy and can be considered to have chronic (cold type) CRPS based on the disease duration. Thus, a limitation of our study is that there may be a referral bias in the study population resulting in a patient sample that may not be completely representative of the general CRPS patient population. Therefore, it is not unlikely that if this study were to be replicated in another setting such as a secondary hospital where patients are seen at an earlier stage and/or with a warm limb, it might return different results. We therefore suggest that future research replicates this study in a primary or secondary care setting. Furthermore, future research should also focus on measuring other inflammatory markers in CRPS, for example, cytokines or other soluble surface molecules secreted from activated immune cells.

Another limitation of our study is that the current sample size was calculated based on the effect size which was derived from our first study in which we investigated whether there was a difference in serum sIL-2R levels between CRPS patients and healthy controls [8]. The observed effect size from this previous study was rather large and may have led to an underestimation of the required sample size for the current study. Therefore, this study may have been underpowered for the primary outcome: the difference between sIL-2R levels in the CRPS group and no CRPS group. Furthermore, we chose not to conduct corrections for multiple testing with regard to the secondary outcomes as it may have barred the discovery of potential associations that could be of interest to explore in future research.

Despite the limitations mentioned above, we believe that the greatest strength of our study is the selection of the study population. All patients included in this study were suspected of having CRPS. Therefore, our no CRPS group consisted of various diseases that can display the same symptoms and signs as CRPS in a limb. Thus, our study design closely reflects clinical practice, especially in a tertiary care setting, and could be used as a model for replication studies.

## 5. Conclusion

In summary, we conclude that serum sIL-2R cannot be used in a tertiary referral setting to differentiate CRPS from other pain conditions of a limb in patients referred with a suspicion of CRPS. Our current findings confirm the findings from our previous study in which serum sIL-2R levels are shown to be higher in CRPS patients than in healthy controls, suggesting a role for pathogenic T-cell activation in CRPS [8].

Although serum sIL-2R may not be useful in establishing the diagnosis CRPS, future studies should focus on replicating this study in a primary and/or secondary care setting and should further focus on exploring the relationship between sIL-2R and (T-cell mediated) disease activity and disease severity in CRPS. These explorations could reveal a possible role for sIL-2R as a biomarker for disease activity and/or severity in CRPS and could further reveal a possible role for sIL-2R as a biomarker for selection of (anti-inflammatory) therapies in CRPS.

## Figures and Tables

**Table 1:** Inclusion and exclusion criteria applied in this study. Patients had to meet both the inclusion criteria and were excluded if they met any of the exclusion criteria.

Inclusion criteria	Exclusion criteria
Age ≥ 18 years	History of an autoinflammatory or autoimmune disease
Only one limb is affected	Current or past (within the last six months) treatment with immunomodulating medication such as steroids or TNF-*α* inhibitors
	Ill in the past two weeks or at the time of visit
	Potential pregnancy or confirmed pregnancy

**Table 2:** Symptoms and signs assessed using the CRPS severity score-Database Form by Harden et al. [15].

Symptoms^∗^^1^	Signs^∗^^2^
Continuing, disproportionate pain	Hyperalgesia to single pinprick
Allodynia or hyperalgesia	Allodynia
Temperature asymmetry	Temperature asymmetry by palpation
Color asymmetry	Color asymmetry
Sweating asymmetry	Sweating asymmetry
Edema	Asymmetric edema
Dystrophic changes	Dystrophic changes
Motor abnormalities^∗^^3^	Motor abnormalities^∗^^4^

^∗^^1^Symptoms as reported by the patient. All symptoms are categorical variables and are registered as absent or present. ^∗^^2^Signs as observed during physical examination by the physician. All signs are categorical variables and are registered as absent or present. ^∗^^3^Motor abnormalities as reported by the patient: weakness, tremor, dystonia, decreased range of motion, and myoclonus. ^∗^^4^Motor abnormalities as observed by the examiner: tremor/myoclonus, dystonia, decreased active range of motion, and weakness.

**Table 3:** Diagnosis and median sIL-2R level per group.

Diagnosis	Total patients per group	Median sIL-2R pg/ml (Q3-Q1)
Healthy controls	101	1515.0 (1880.0-1150.0)
CRPS group	23	2809.0 (3913.0-1589.0)
No CRPS group	29	3654.0 (4429.0-2095.5)
Neuropathic pain syndromes^∗^^1^ (*n*, % no CRPS group)	7 (24.1)	4170.0 (5203.0-2050.0)
Myofascial pain syndromes^∗^^2^ (*n*, % no CRPS group)	17 (58.6)	3529.0 (4253.5-2150.5)
Inflammation^∗^^3^ (*n*, % no CRPS group)	2 (6.9)	N/A
Unknown^∗^^4^ (*n*, % no CRPS group)	3 (10.3)	N/A

^∗^^1^Neuropathic pain syndromes: peripheral neuropathy (*n* = 5); cervical dermatomal pain (*n* = 1); radicular pain (*n* = 1). ^∗^^2^Myofascial pain syndromes: postfracture pain and osteoarthritis (*n* = 1); osteoarthritis (*n* = 1); disuse (*n* = 1); myalgia (*n* = 1); disability and impairment of hand related to fracture as diagnosed by plastic surgeon (*n* = 2); shin splints (*n* = 1); subacromial pain syndrome (*n* = 1); unspecified pain of the shin (*n* = 1); suspected patellofemoral pain syndrome (*n* = 1); suspected clenched fist syndrome (*n* = 1); pain related to healing process after trauma (*n* = 4); postsurgical pain (*n* = 2). ^∗^^3^Inflammation: osteomyelitis (*n* = 1); arthritis of the wrist (*n* = 1). Median sIL-2R levels were not calculated due to the size of the group. ^∗^^4^Median sIL-2R levels were not calculated due to the size of the group.

**Table 4:** Patient demographics and general characteristics of the no CRPS and CRPS group.

Demographics and characteristics	No CRPS (*n* = 29)	CRPS (*n* = 23)	Significance
Age in years (median, (Q3-Q1))	43.0 (55.5-27.5)	37.0 (55.0-28.0)	NS
Duration of disease in months (median, (Q3-Q1))	20.0 (36.0-8.5)	26.0 (81.0-14.0)	NS
Gender			NS
Male (*n*, %)	10 (34.5)	4 (17.4)	
Female (*n*, %)	19 (65.5)	19 (82.6)	
Affected limb			NS
Right upper limb (*n*, %)	6 (20.7)	5 (21.7)	
Left upper limb (*n*, %)	5 (17.2)	4 (17.4)	
Right lower limb (*n*, %)	6 (20.7)	4 (17.4)	
Left lower limb (*n*, %)	12 (41.4)	10 (43.5)	
Precipitating injury			NS
Trauma	13 (44.8)	11 (47.8)	
Operation	11 (37.9)	9 (39.1)	
Spontaneous	5 (17.2)	0	
Other	0	2 (8.7)	
Unknown	0	1 (4.3)	

**Table 5:** Medications being used at the time of visit at the outpatient clinic center.

Medication	No CRPS (*n* = 29)	CRPS (*n* = 23)	Significance
Paracetamol (*n*, %)	10 (34.5)	9 (39.1)	NS
NSAIDs^∗^^1^ (*n*, %)	10 (34.5)	5 (21.7)	NS
Opioids (*n*, %)	5 (17.2)	8 (34.8)	NS
Antidepressants (*n*, %)	3 (10.3)	6 (26.1)	NS
Antiepileptics (*n*, %)	3 (10.3)	6 (26.1)	NS
Calcium channel blockers (*n*, %)	1 (3.4)	2 (8.7)	NS
Phosphodiesterase-5 inhibitor (*n*, %)	0	0	N/A
Vitamin C (*n*, %)	6 (20.7)	3 (13.0)	NS
Fluimucil or N-acetyl cysteine (*n*, %)	0	1 (4.3)	N/A
DMSO^∗^^2^ (*n*, %)	2 (6.9)	0	N/A

^∗^^1^NSAIDs: nonsteroidal anti-inflammatory drugs. ^∗^^2^DMSO: dimethylsulfoxide cream.

**Table 6:** CRPS severity score-Database Form: presence of symptoms and signs of CRPS in each group.

Symptoms	No CRPS (*n* = 29)	CRPS (*n* = 23)	Significance
NRS at time of visit (median, Q3-Q1)	7.0 (8.0-3.0)	7.0 (8.0-6.0)	NS
NRS 24 hours before visit (median, Q3-Q1)	7.5 (8.0-6.3)	8.0 (8.0-7.0)	NS
Continuing pain (*n*, %)	18 (62.1)	23 (100)	*p* = 0.001
Allodynia and/or hyperalgesia	27 (93.1)	23 (100)	NS
Allodynia	14 (48.3)	17 (73.9)	NS
Hyperalgesia	24 (82.8)	23 (100)	NS
Temperature asymmetry	27 (93.1)	20 (87.0)	NS
Affected side warmer	11 (37.9)	7 (30.4)	NS
Affected side colder	9 (31.0)	5 (21.7)	NS
Affected side warm/cold	7 (24.1)	8 (34.8)	NS
Color asymmetry	23 (79.3)	23 (100)	*p* = 0.028
Red	14 (48.3)	13 (56.5)	NS
Blue	5 (17.2)	8 (34.8)	NS
Other color	12 (41.4)	14 (60.9)	NS
Sweating asymmetry	12 (41.4)	14 (60.9)	NS
Edema	24 (82.8)	21 (91.3)	NS
Dystrophic changes	15 (51.7)	17 (73.9)	NS
Nails	10 (34.5)	12 (52.2)	NS
Hair	8 (27.6)	11 (47.8)	NS
Skin	6 (20.7)	10 (43.5)	NS
Motor abnormalities	29 (100)	23 (100)	N/A
Weakness	25 (86.2)	22 (95.7)	NS
Tremor	15 (51.7)	13 (56.5)	NS
Dystonia	13 (44.8)	10 (43.5)	NS
Decreased AROM	20 (69.0)	22 (95.7)	*p* = 0.030
Myoclonus	4 (13.8)	9 (39.1)	NS
Signs	No CRPS (*n* = 29)	CRPS (*n* = 23)	Significance
Hyperalgesia to pinprick	11 (37.9)	17 (73.9)	*p* = 0.013
Allodynia	18 (62.1)	22 (95.7)	*p* = 0.007
Light touch	6 (20.7)	19 (82.6)	*p* < 0.001
Deep joint pressure	9 (31.0)	18 (78.3)	*p* = 0.002
Vibration	8 (27.6)	14 (60.9)	*p* = 0.021
Cold	2 (6.9)	11 (47.8)	*p* = 0.002
Heat	3 (10.3)	11 (47.8)	*p* = 0.004
Temperature asymmetry on palpation	2 (6.9)	8 (34.8)	*p* = 0.015
Affected side cooler	1 (3.4)	8 (34.8)	*p* = 0.007
Affected side warmer	1 (3.4)	0	NS
Color asymmetry	4 (13.8)	12 (52.2)	*p* = 0.006
Red	3 (10.3)	9 (39.1)	*p* = 0.021
Blue or pale	3 (10.3)	4 (17.4)	NS
Mottled	0	4 (17.4)	N/A
Scar	0	0	N/A
Sweating asymmetry	1 (3.4)	6 (26.1)	*p* = 0.035
Increased on affected side	1 (3.4)	6 (26.1)	*p* = 0.035
Decreased on affected side	0	0	N/A
Asymmetric edema	1 (3.4)	6 (26.1)	*p* = 0.035
Dystrophic changes	4 (13.8)	7 (30.4)	NS
Nails	1 (3.4)	5 (21.7)	NS
Hair	3 (10.3)	2 (8.7)	NS
Skin	1 (3.4)	4 (17.4)	NS
Motor abnormalities affected side	20 (69.0)	21 (91.3)	NS
Tremor or myoclonus	2 (6.9)	6 (26.1)	NS
Dystonia	2 (6.9)	7 (30.4)	NS
Decreased AROM	16 (55.2)	17 (73.9)	NS
Weakness 1/5^∗^^1^	0	3 (13.0)	N/A
Weakness 2/5^∗^^2^	0	5 (21.7)	N/A
Weakness 3/5^∗^^3^	4 (13.8)	5 (21.7)	NS
Weakness 4/5^∗^^4^	12 (41.4)	7 (30.4)	NS
CRPS severity score (mean, sd)	8.1 (1.9)	11.4 (2.2)	*p* <0.001

^∗^^1^Weakness 1/5: flicker of movement. ^∗^^2^Weakness 2/5: movement with gravity. ^∗^^3^Weakness 3/5: movement against gravity. ^∗^^4^Weakness 4/5: weak.

## Data Availability

The data used to support the findings of this study are restricted by the Medical Ethics Committee of Erasmus MC University Rotterdam and the General Data Protection Regulation of the EU in order to protect patient privacy.
